# Investigation of the Precision in Geodetic Reference-Point Positioning Because of Temperature-Induced Pillar Deflections

**DOI:** 10.3390/s19163489

**Published:** 2019-08-09

**Authors:** Robert Močnik, Božo Koler, Dejan Zupan, Tomaž Ambrožič

**Affiliations:** 1 Mariborski vodovod d.d., Jadranska cesta 24, 2000 Maribor, Slovenia; 2 Faculty of Civil and Geodetic Engineering, University of Ljubljana, Jamova 2, 1000 Ljubljana, Slovenia

**Keywords:** reinforced concrete pillar, based reference point, influence of temperature, calculated displacement, measured displacement, temperature distribution

## Abstract

To perform geodetic measurements of displacements of the ground and manmade constructions, stabilised reference points are needed from which control points on the object or its surroundings could be measured. Reference points are most commonly stabilised with reinforced concrete pillars; however, they are not always constructed in an appropriate manner. The influence of temperature variation within a pillar on the position of the fixed screw for forced centring is not negligible and should be considered when performing precise measurements. In this research paper, the displacement of a pillar was calculated as a result of the temperature changes in the pillar, and then an experiment was performed in which the pillar was heated from one side, and the horizontal displacement of the fixed screw for forced centring was measured. Both, calculations and measurements, show that at a temperature difference of 16.2 °C, the fixed screw on a 1.5 m high pillar moves by approximately 1 mm, which is a displacement that should be taken into account in precise measurements.

## 1. Introduction

Geodetic positioning necessary for various applications is based on reference points, most of which are in the form of concrete pillars about 1.5 m high. Most reinforced concrete pillars have a round cross-section with a diameter ranging between 20 cm and 60 cm with fixed screws for forced centring or with ground points and an eccentric standing point from which the measurements are conducted [1]. The pillars can also have a rectangular cross-section or be shaped as a sliced pyramid. Sometimes, the pillars are surrounded by a protective exterior layer. In this case, the space between the pillar and the protective layer should be filled with heat insulation to reduce the influence of the changes in temperature that occur naturally from solar radiation. The pillars should be stable, and the reference point coordinates should be determined with sub-millimetre precision; otherwise, their positioning error may influence the coordinates of the control points. Reference and control points could be connected to the geodetic network using precise geodetic measurements. By using a precision geodetic instrument and accessories and taking into account all the influences, it is possible in such a network to determine the displacement of the control points on an object with sub-millimetre precision.

**Figure 1:**
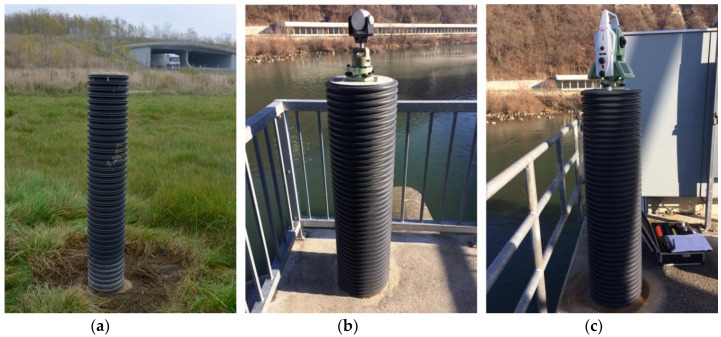
Examples (**a**–**c**) of inappropriately chosen reference points; the pillars are thin and wrapped in black PVC tubes.

Some of the stabilisation of reference points with reinforced concrete pillars is not carried out with due attention. They are often positioned on unstable grounds, and, in an attempt to lower the costs, they usually fail to have an appropriate base or are wrapped in an inappropriate layer (black PVC tubes—Figure 1). The relatively small cross-sections and the dark—most often black—colour of the tubes can represent a serious problem, especially when a tube is exposed to direct sunlight in otherwise low environment temperatures. The great emission factor of the surrounding black tube leads to localised temperature changes within the reinforced concrete pillar. When one side of the pillar is exposed to solar radiation, these localised temperature changes within the reinforced concrete pillar cause the sunlit part to expand, which consequentially results in bending of the pillar. As the pillar bends, vertical and horizontal displacements of the fixed screws for forced centring are observed. These displacements are not necessarily negligible and might significantly influence the stability of the reference point. Although the problem has already been recognised, such sources of error are still being encountered, even in newly-built reference points.

The goal of this research is to establish experimental and theoretical estimations of temperature-based displacements of pillars, sometimes used for reference points. The results were compared according to the linear elastic mechanical model of the pillar, which is easy to understand and used for further predictions and experimental results obtained in a controlled laboratory environment. This paper focuses merely on the horizontal displacements that are dominant when the strains in the pillar are small. An additional reason for addressing the horizontal displacement is that these pillars are usually reference points in horizontal geodetic networks.

As the stabilisation of reference points with reinforced concrete is most commonly used in precise geodetic measurements, a 1 mm displacement at the top of the pillar represents a large systematic error. Such displacements are realistically expected when these pillars bend due to temperature. As the reference point in the horizontal geodetic network is treated as a given point, its displacement influences the calculated position of the control points. This could lead to the incorrect conclusion that there was a displacement of the control point on the object, even though its displacement was merely the result of an error due to the temperature changes of the pillar. In order to describe the displacements of the control points with sufficient accuracy, it is necessary to take into account the possibility of the pillar displacement due to the temperature changes in the pillar.

## 2. Methods

### 2.1. Previous Studies on the Subject and Related Work

The problem of temperature influence on reference points for precise geodetic measurements has already gained some attention among researchers. Lindberg and Lilje [2] calculated and measured the deformation of the 3 m high reference point in a SWEPOS GNSS network. The horizontal displacement ranged from 0.18 mm at a temperature difference of 1 K to 1.80 mm at a temperature difference of 10 K. Lehner [3] and Hass et al. [4] analysed various ways of stabilising reference points with respect to deformations that occur as a result of solar radiation, temperature variations, and wind. For different shapes of 3.2 m high reference points, horizontal displacements of 0.9 mm to 4.0 mm were obtained at a temperature difference of 28 °C [3]. Gerhatova et al. [5,6] also tested the deformations of reference points as a result of direct solar exposure and proved the correlations with daily temperature and solar radiation changes. The concrete pillar, 3 m high and 1 m in diameter, moved up to 1.16 mm at a temperature difference of 18 °C in the east-west direction and up to 0.94 mm in the north-south direction. The concrete pillar, 1.21 m in height and 0.40 m in diameter, moved up to 1.17 mm at a temperature difference of 28 °C in the east-west direction and up to 0.76 mm in the north-south direction. The displacement of the concrete pillar of the dimension 0.75 × 0.50 m in both directions did not exceed 0.04 mm at a temperature difference of 12 °C. The horizontal displacement of the reference point due to solar radiation and the related temperature changes were also measured with an inclinometer and control measurements by Kopačik et al. [7] (the maximum displacement vector was 1.2 mm), and Lipták [8] who took this displacement into account in all future calculations. Santamaría-Gómez [9] also ascertained that the thermo-elastic expansion of the reference points and concrete buildings, driven by solar heating, is the likely the origin of the oscillation in the baseline length (the annual amplitude is 1.7 mm at an annual temperature difference of 10 °C). Filipiak-Kowszyk et al. [10] determined the displacement of an instrument stand (up to 2.7 mm in 24 h), which was located on the roof cover of the Forest Opera in Sopot, during monitoring of the movement of control points.

### 2.2. Theoretical Model

**Figure 2:**
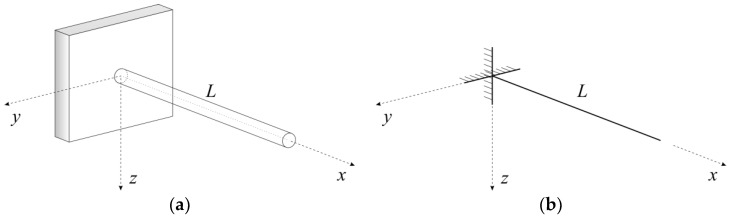
(**a**) A three-dimensional body; (**b**) A numerical model of the beam.

The elementary equations of a clamped beam under a linearly distributed temperature are summarised below. For the purposes of this research, it was assumed that the axis of the beam is straight and that it lies along the *x*-axis in a Cartesian coordinate system (Figure 2).

The deformation of such a beam could be well described with a single non-zero component of the strain tensor:

(1) $$\varepsilon_{xx}=\varepsilon_{xx}\left(x,y,z\right),$$

while the remaining components could be neglected.

The strains are then related to the displacements of the reference axis:

(2) $$u=u\;e_{x}+v\;e_{y}+w\;e_{z},$$

where u denotes the axial displacement, while v and w are the lateral displacements. The following kinematic equation of a beam was then obtained directly from the definition of the strain tensor:

(3) $$\varepsilon_{xx}=\frac{du}{dx}-y\frac{d^{2}v}{dx^{2}}-z\frac{d^{2}w}{dx^{2}}.$$

For an ideal linear-elastic, homogenous, isotropic material the following holds true [11]:

(4) $$\varepsilon_{xx}\left(x,y,z\right)=\alpha_{T}\;∆T\left(x,y,z\right),$$

where $\alpha_{T}$ denotes the linear expansion coefficient of the material and ΔT(x,y,z) the change in temperature at the point (x,y,z) of the beam. Here, a simplified temperature distribution has been assumed, where the temperature changes are distributed linearly along the cross-section leading to:

(5) $$∆T\left(x,y,z\right)=∆T_{x}\left(x\right)+∆T_{y}\left(x\right)\;y+∆T_{z}\left(x\right)\;z,$$

where the coefficients $\Delta T_{x}$, $\Delta T_{y}$, and $\Delta T_{z}$ are dependent only on the arc-length parameter of the axis of the beam. From Equations (3) and (4), and taking into account Equation (5), results in:

(6) $$\frac{du}{dx}=\alpha_{T}\;∆T_{x},\;-\frac{d^{2}v}{dx^{2}}=\alpha_{T}\;∆T_{y}\;\mathrm{and}\;-\frac{d^{2}w}{dx^{2}}=\alpha_{T}\;\Delta T_{z}.$$

Here a simple cantilever beam has been considered with a constant cross-section and with a constant temperature load along the y direction ($\Delta T_{y}=0\;K$).

**Figure 3:**
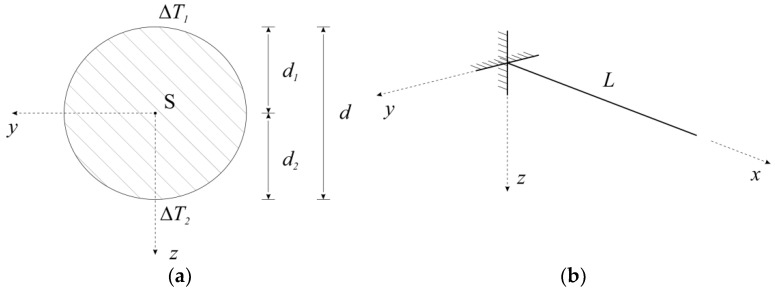
(**a**) The cross-section of the beam; (**b**) A computational model of the cantilever.

With regard to Figure 3, it could be stated that for $z=-d_{2},$ the change in temperature is $\Delta T=\Delta T_{2}$ and for $z=d_{1}$, the change in temperature is $\Delta T=\Delta T_{1}$. Thus $\Delta T_{1}=\Delta T_{x}-d_{1}\;\Delta T_{z}$ and $\Delta T_{2}=\Delta T_{x}+d_{2}\;\Delta T_{z}$. The difference in temperature is:

(7) $$\Delta T_{2}-\Delta T_{1}=d_{2}\;\Delta T_{z}+d_{1}\;\Delta T_{z}=\left(d_{1}+d_{2}\right)\Delta T_{z}.$$

leading to

(8) $$\Delta T_{z}=\frac{\Delta T_{2}-\Delta T_{1}}{d}.$$

The lateral displacement is then obtained by solving differential Equation (6) and taking into account that the beam is clamped (w(0) = 0 , ${\frac{dw}{dx}|}_{x=0}\;=\;0$). This leads to the simple formula:

(9) $$w\left(x\right)=-\alpha_{T}\;\Delta T_{z}\frac{\;x^{2}}{2}.$$

Consideration of Equations (8) and (9) results in:

(10) $$w\left(x\right)=\frac{\alpha_{T}\;\left(\Delta T_{1}-\Delta T_{2}\right)\;x^{2}}{2\;d}.$$

The authors of the research were particularly interested in free-end displacements, when $\Delta T_{2}=0\;K$. They read:

(11) $$w\left(L\right)=\frac{\alpha_{T}\;\Delta T_{1}\;L^{2}}{2\;d},$$

which could be taken as a simple engineering formula for the estimation of the temperature-related displacements of the standing point for the measuring equipment.

For the purpose of validation, the following is then expressed:

(12) $$w=k\;\Delta T_{1},$$

which gives a formula for the evaluation of the temperature expansion coefficient:

(13) $$\alpha_{T}=\frac{2\;d\;k}{L^{2}}.$$

In accordance with the law of transformation of variance/covariance matrices, this now enables the standard deviation of the linear temperature expansion coefficient to be calculated with the following equation:

(14) $$\sum_{\alpha_{T}\alpha_{T}}=\left[\frac{\partial \alpha_{T}}{\partial k}\right]\;\left[\sigma_{k}^{2}\right]\;{\left[\frac{\partial \alpha_{T}}{\partial k}\right]}^{T}=\left[\sigma_{\alpha_{T}}^{2}\right],$$

where

(15) $$\frac{\partial \alpha_{T}}{\partial k}=\frac{2\;d}{L^{2}}.$$

The final result from Equations (13) and (14) is:

(16) $$\sigma_{\alpha_{T}}=\frac{2\;d}{L^{2}}\sigma_{k}.$$

### 2.3. Constructing the Reinforced Concrete Pillar That Could Serve as a Reference Point

**Figure 4:**
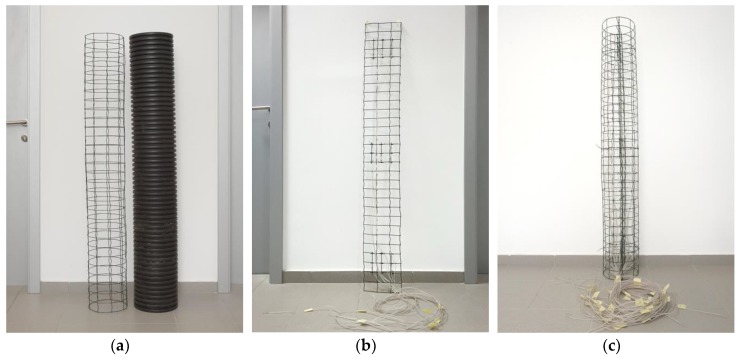
(**a**) The reinforcement rolled in the form of a cylinder and the PVC tube; (**b**) The reinforcement in the form of a plain mesh with the attached temperature sensors; (**c**) PVC tube and temperature sensors ready to be covered in concrete.

When constructing the pillar, a ribbed tube was used made of plastic material (in the continuation PVC tube) with an outer diameter of 250 mm and an inner diameter of 217 mm; the height of the pillar was 1510 mm. A welded reinforcement was rolled in the form of a cylinder (Figure 4a) so that it fits inside the PVC tube. An additional in-place reinforcement (Figure 4b) was placed in the middle of the PVC tube, which was positioned perpendicular to the heat source. This reinforcement was used to attach temperature sensors (thermo-elements). Temperature sensors were also attached to the circumferential reinforcement in three cross-sections (Figure 4c).

**Figure 5:**
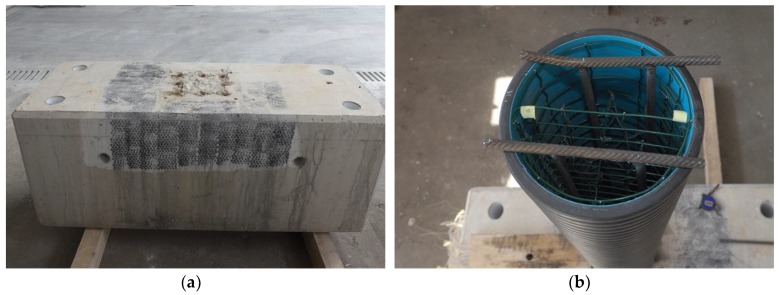
(**a**) A concrete block; (**b**) Tube ready for pouring concrete.

The concrete base of the pillar is depicted in Figure 5a. The ribbed reinforced rods, measuring 12 mm in diameter, were anchored into the drilled holes within the concrete block with the chemical anchoring system. Transversal rods used to stabilise the PVC tube while pouring the concrete were welded on top of the anchoring reinforcement rods (Figure 5b).

### 2.4. Measuring Equipment

**Figure 6:**
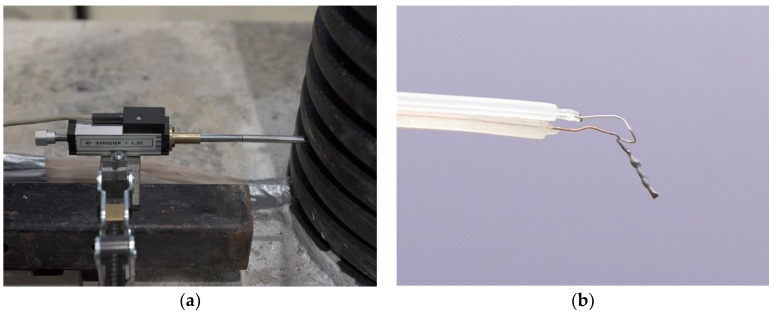
(**a**) A Novotechnik TR25 potentiometric position transducer; (**b**) Welded thermocouple wire.

The displacements of the pillar were measured with a Novotechnik TR25 potentiometric position transducer (Novotechnik U.S. Inc., Southborough, MA, USA) (Figure 6a), which was attached to an HBM MGCplus AB22A amplifier (Hottinger Baldwin Messtechnik GmbH, Darmstadt, Germany), which, together with the HBM Catman AP V3.5.1 software, enabled the capture, analysis, and visualisation of the data. According to the manufacturer, the measurement accuracy is within 0.002 mm [12].

The temperature within the pillar was measured using a welded thermocouple wire (Figure 6b). The Dewetron system for data capturing and the Agilent VEE Pro application were used to gather data from the thermocouple wire. The measurements are precise up to 0.3 K.

**Figure 7:**
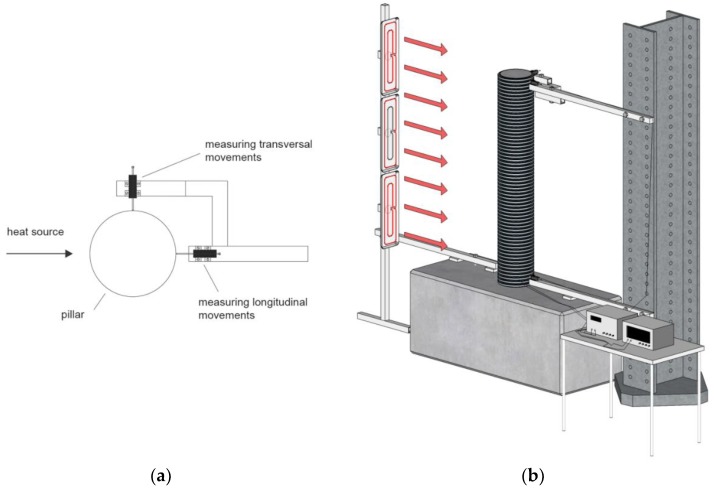
(**a**) The position of the displacement sensor at the top of the pillar; (**b**) The scheme of the experiment (the red arrows indicate the direction of the heat flow).

A steel bar vertical I-profile was used between the sensors, which served as a reference surface for measuring the displacements (Figure 7a). The steel bar was isolated to reduce the influence of the temperature. Three sensors for measuring the displacements were used: two at the top of the pillar, at the height of 1500 mm, and one at the bottom. One of the sensors at the top was attached in the direction of the applied heat. The second sensor at the top was perpendicular to the heat flow (Figure 7b). The sensor at the bottom was attached at the height of 70 mm, and parallel to the heat flow. The data obtained from this sensor were used as control data.

**Figure 8:**
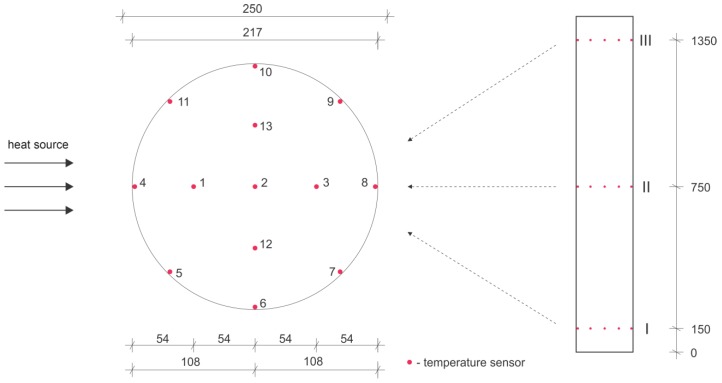
The position of the temperature sensors within the reinforced concrete pillar, cross-section (**left**) and longitudinal cross-section (**right**).

The temperature sensors were located in three cross-sections (I, II, and III) (Figure 8, right), at heights of 150 mm, 750 mm, and 1350 mm, respectively. Each cross-section held 13 temperature sensors (Figure 8, left).

## 3. Results

During the experiment, the displacements of the pillar were measured at the three points simultaneously. The positions of these points were explained in detail in the previous section. During the experiment, negligible displacements were observed at the top and the bottom in the direction perpendicular to the heat source. Thus, they are not shown here. The dominant displacements were found in the direction of the heat flow.

The temperature was measured at 39 points (all equipped with temperature sensors), positioned at three cross-sections (marked I, II, and III) and located at various heights within the pillar. The sensors were calibrated according to the initial uniform temperature conditions.

**Figure 9:**
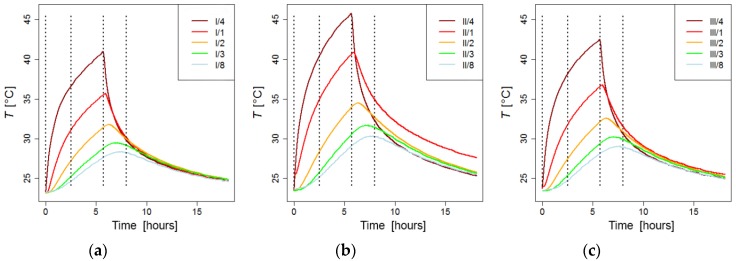
The temperature at discrete points of the concrete pillar as a function of time; the dashed lines denote the discrete times where the temperature distribution is presented over the cross-section: (**a**) Cross-section I; (**b**) Cross-section II; (**c**) Cross-section III.

Figure 9 shows the changes in temperature at cross-sections I, II, and III with respect to time. The changes in the temperature are shown for sensors 1–4 and 8 for each of the cross-sections. These sensors are located in the mid-plane parallel to the heat flow (Figure 8). In the figures, they are marked so that the first number (I, II, or III) denotes the cross-section while the second number (1–4, 8) denotes the position of the sensor within the individual cross-section. The sensors denoted as I/4, II/4, and III/4 are the closest to the heat source, while the sensors denoted as I/8, II/8, and III/8 are the furthest away (on the back of the pillar).

According to theoretical results, the temperatures at the mid-plane of the cross-sections are crucial. Figure 9 shows that the temperature at the front of the pillar (sensors I/4, II/4, and III/4) rises until the moment the heat source is turned off (5.7 h after the heating was started). These sensors show an instantaneous drastic fall in temperature as soon as the heat source is turned off, followed by a more gradual decline in temperature. It was observed that the remaining sensors (at points 1, 2, 3, and 8), in all cross-sections (I, II, and III)), show that the temperature continues to rise for a while after the heat source is turned off and only then begins to decline. It takes longer for the temperature to start declining at the sensors that are further away from the front of the pillar. By increasing the distance, the delay is also increased.

**Figure 10:**
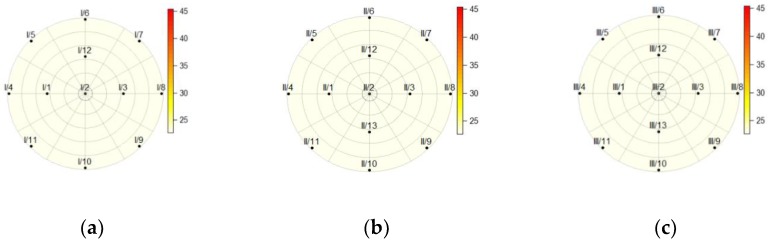
The temperature within the pillar at a height of (**a**) 150 mm; (**b**) 750 mm; (**c**) and 1350 mm before the heat source was turned on (*t* = 0 h).

**Figure 11:**
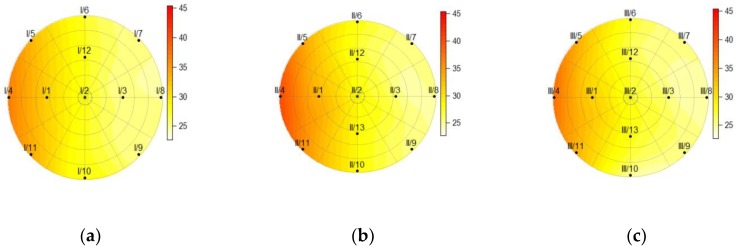
The temperature within the pillar at a height of (**a**) 150 mm; (**b**) 750 mm; (**c**) and 1350 mm, 2.5 h after the heat source was turned on.

**Figure 12:**
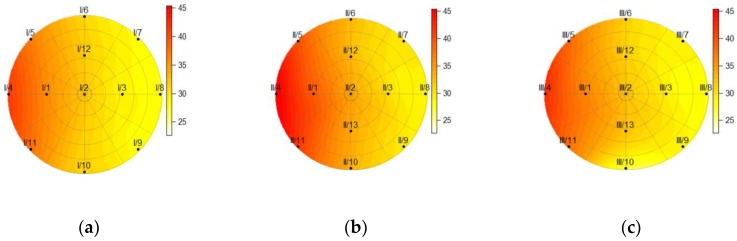
The temperature within the pillar, at a height of (**a**) 150 mm; (**b**) 750 mm; (**c**) and 1350 mm, 5.7 h after the heat source was turned on (before it was turned off).

**Figure 13:**
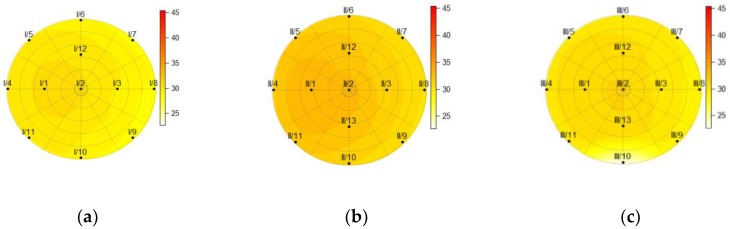
The temperature within the pillar at a height of (**a**) 150 mm; (**b**) 750 mm; (**c**) and 1350 mm, 2.3 h after the heat source was turned off.

Figure 10, Figure 11, Figure 12 and Figure 13 show the temperatures within the three cross-sections of the pillar (at heights of 150, 750, and 1350 mm) at various discrete times. The data obtained from the measurements revealed that the sensor at point 13 on cross-section I was damaged; thus all the data obtained from this sensor were eliminated from the calculations.

After 2.5 h of heating, the distribution of temperatures throughout the cross-sections reveals that the temperature on the back side of the pillar (I/8, II/8, and III/8) remained unchanged (Figure 11).

The charts of the temperature distributions at cross-sections I, II, and III (Figure 11 and Figure 12) reveal that the heating across the entire height of the pillar was not homogenous, as cross-section II shows higher temperatures than cross-sections I and III. It was hard to achieve perfect homogeneity using the equipment available. Figure 12 shows the cross-sections at the time the heat source was turned off. It is clearly visible that the temperature also rose on the back side of the pillar (the sensor at point 8) in all three cross-sections (I, II, and III).

Figure 13 reveals that the temperature at point 8 on cross-section II had raised slightly two hours after the heat source was turned off. The temperatures at the cross-sections also showed that the pillar cooled down faster at the bottom where it was fixed on a massive concrete block.

**Figure 14:**
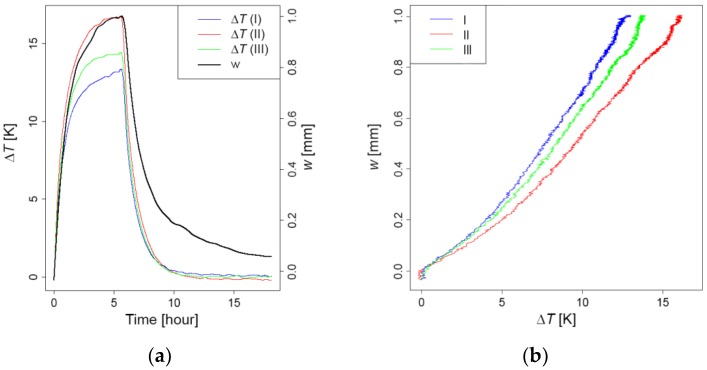
(**a**) The time response of the temperature difference and the top deflections; (**b**) The relation between the top deflections and the temperature difference.

As no mechanical loads were applied to the pillar, the deformation of the pillar was influenced mainly by the changes in the temperature (see Equation (11)). In order to define the changes in temperature, measurements were used from sensors located at points 4 and 8. Figure 14a shows the time response of the difference between the temperatures at the front (point 4) and at the back (point 8) of the cross-section at all three cross-sections and the displacements at the top of the pillar. Figure 14b shows the relation between the temperature difference and the lateral deflections at the top in the direction of heat radiation. It could be seen that the relation between the horizontal displacements and the temperature difference is close to linear. The lateral deflections of the pillar were negligible; the maximum measured value was 0.03 mm.

**Figure 15:**
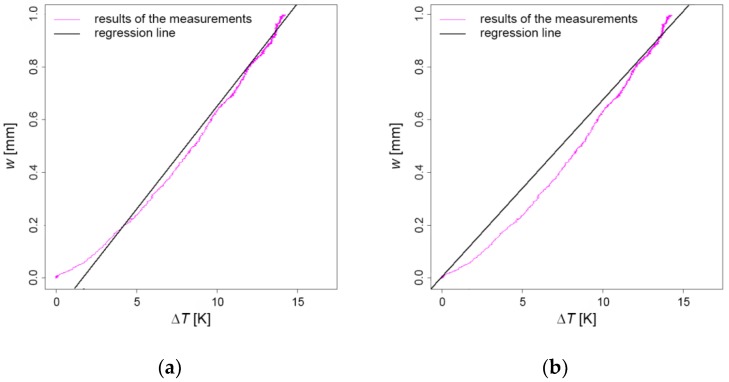
(**a**) The regression line relating the temperature differences and the top deflections; (**b**) The regression line additionally assuming the intercept at zero.

Based on the data obtained, the regression line for the correlation between the temperature difference and the top deflections of the pillar was evaluated. The average temperature difference was used from all three cross-sections (I, II, and III) as the input data. Figure 15a shows the obtained regression line. The regression line formula is $w=-0.177\;\mathrm{mm}+0.078\;{\mathrm{mm}\;K}^{-1}\cdot ∆T_{1}$, which shows small but evident discrepancies at low temperatures.

Since it was assumed that there is no displacement at the initial time, the constant of the equation for the regression line was set at zero (Figure 15b). This resulted in $w=+0.064\;{\mathrm{mm}\;K}^{-1}\cdot ∆T_{1}$, where the standard deviation of the inclination k was $\sigma_{k}=9.901\cdot 10^{-5}\;{\mathrm{mm}\;K}^{-1}$.

It is evident that the linear model cannot fully describe the behaviour of the pillar; however, it still provides very satisfactory results. Prior to the introduction of more sophisticated models, which in the authors’ view are not needed, additional attention should be dedicated to sensor calibration and measurement of heat source uniformity.

## 4. Analysis and Discussion

After evaluating the data of Equation (13): L=1500 mm, d=217 mm and $k=0.064{\;\mathrm{mm}\;K}^{-1}$, this resulted in 1.241 × 10^−5^ K^−1^,which is taken as the average value for further analysis.

The standard deviation of the linear temperature expansion coefficient based on the estimate of $\sigma_{k}$ from the linear regression in the previous section and Equation (16) is estimated to be 0.002 × 10^−5^ K^−1^.

This result is then used to estimate the 95 per cent confidence interval of the median value of the linear temperature expansion coefficient $[\bar{\alpha_{T}}-1.96\cdot \sigma_{\alpha_{T}},\bar{\alpha_{T}}+1.96\cdot \sigma_{\alpha_{T}}]$. This produces $\alpha_{T}\in [1.237,1.244]\cdot 10^{-5}\;K^{-1}$.

Thus, the estimated interval is in accord with the known thermal properties of concrete obtained from larger samples and, thus, confirms the correctness of the results [13].

From the theoretical and experimental approach, the authors of this research evaluated the realistic temperature-dependent deflections at the top of the concrete reference point. At the temperature difference 13.0 °C at cross-section I (at the height of the reference point 150 mm), 16.2 °C at cross-section II (at the height of the reference point 750 mm), and 13.9 °C at cross-section III (at the height of the reference point 1350 mm), the largest measured absolute displacement at the top of the reference point was 0.99 mm.

Based on both the theoretical model and experimental data, it could be confirmed that the temperature has a significant influence on the reference point when performing precise measurements. Due to the error, which emerges as a result of the change in temperature within the reference point, it is possible to reach the wrong conclusion regarding the displacement of the control point. In practice, a combination of errors occurs, which can lead to significant errors when evaluating the overall stability of the object. In the evaluation process of the displacement or the stability of important objects, it is thus essential to perform all measurements at temperatures that are as similar as possible to those during the initial measurements.

**Figure 16:**
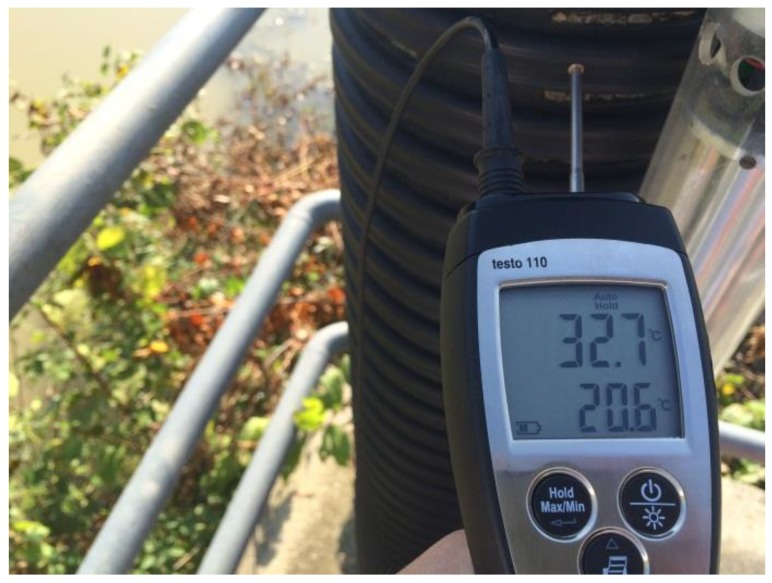
The measured temperatures on the sunny and shady sides of the reference point; the upper value shows the temperature on the sunny side, while the lower value shows the temperature on the shady side.

When measuring the geodetic network, which is located at the hydropower plant on the Sava river, a contact thermometer was used to measure the temperatures on the sunny and shady sides of the reference point, without a previous plan (Figure 16). The photo shows that the results were not that far from the assumptions of this study.

## 5. Conclusions

The authors have presented the influence of thermal loads on concrete reference points for precise geodetic measurements. It was found that this influence is not negligible. Displacements were obtained theoretically using a beam model and experimentally. This analysis was performed in a controlled laboratory environment. The experiment confirmed the matching of the measured displacement of the pillar under consideration with a calculated displacement of 0.99 mm per 1.5 m high and a temperature difference of 16.2 °C. The calculated thermal expansion coefficient (1.241 ± 0.002) × 10^−5^ K^−1^ is exactly the same as the data from the literature.

In order to discover how such a reference point would behave in natural conditions, it would be necessary to stabilise such a reference point in the field and monitor it there. This would require the reference point to be equipped in the natural environment with a system for measuring displacements over a longer period of time. The ideal scenario would be to monitor the reference point and measure its temperature throughout the year. Due to the complex conditions that this would require; however, as well as the expensive instruments that would be required, it is extremely hard to perform such an experiment.

The influence of the changes in temperature could be reduced by designing measurements in conditions that would lower the impact of the temperature changes within the reference point (for instance performing measurements only when cloudy, painting the reference point in a light colour, using a reference point with a layer of heat insulation, or the better design of a reference point, etc.).
